# Virological non-suppression and associated factors among adult patients receiving antiretroviral therapy at selected health facilities in uMgungundlovu district of KwaZulu Natal, South Africa: a cross-sectional study

**DOI:** 10.11604/pamj.2024.47.96.42338

**Published:** 2024-02-29

**Authors:** Slindile Zondi, Lindiwe Cele, Mmampedi Mathibe, Mabina Mogale

**Affiliations:** 1 Department of Public Health, Sefako Makgatho Health Sciences University, Ga-Rankuwa 0208, South Africa,; 2 Epidemiology and Biostatistics Unit, Department of Public Health, Sefako Makgatho Health Sciences University, Ga-Rankuwa 0208, South Africa

**Keywords:** ART patients, viral suppression, virological non-suppression

## Abstract

**Introduction:**

virological non-suppression is not only associated with increased risk of transmission of the Human Immunodeficiency virus (HIV) to others; perinatally and sexually, but it also decreases the life expectancy among the individuals who are on antiretroviral therapy (ART). This study sought to determine the level of virological non-suppression among ART patients from selected health facilities of a sub-district in uMgungundlovu district. This sub-district has high HIV transmission rates in KwaZulu Natal (KZN) and had one of the highest HIV prevalence in the district in 2018; population weighted HIV prevalence of 36.3% among men and women aged 15-49 years old, which was twice the average national prevalence of 18.8%.

**Methods:**

this descriptive, cross-sectional, and quantitative study was conducted among participants who were HIV-positive, 18 years old and above, and initiated on ART between January 2017 and January 2019 at selected PHC facilities of Vulindlela sub district. Health facility treatment registers, patient medical files and face-to-face interviews were used to collect the data and these were captured onto an Excel spreadsheet, cleaned, coded before importation into Epiinfo 17 for statistical analyses. Logistic regression analyses were conducted to investigate the factors associated with virological non-suppression.

**Results:**

the study found a majority of participants were females (240/401 (60%)). The mean age of the participants was 38.1 (SD=11.2), with most participants who were between the ages of 29 and 39 years old (167 (41.7%)). Virological non-suppression was observed among 10% (40/401) of participants. The odds of virological non-suppression were higher among participants who were married (aOR 4.76, 95% CI 1.49-15.19; p=0.008).

**Conclusion:**

a virological non-suppression of 10% translates to viral suppression of 90%, which is below the target of UNAIDS 95-95-95 strategy. Hiding and skipping medication indicate how non-disclosure continues to hinder HIV treatment adherence. High odds of virological non-suppression among married participants indicate non-disclosure of the positive HIV status, or lack in spousal support.

## Introduction

Virological non-suppression among HIV-positive patients who are on antiretroviral therapy (ART) is associated with increased transmission of the HIV virus to others via perinatal and sexual routes [1]. On the contrary, ART patients who are virally suppressed have an increased likelihood of living long and healthy lives [2]. As a standardized indicator of HIV treatment and prevention success towards ending the HIV epidemic by 2030, viral suppression provides the benchmark for monitoring global targets [3]. Many countries are yet to achieve this target, as the global HIV statistics indicate that only 71% of the 76% global People living with HIV/AIDS (PLWHA) who were accessing ART in 2022, achieved viral suppression [4].

Suboptimal viral suppression rates are seen mostly in countries of the sub-Saharan Africa (SSA), including South Africa, a country with one of the largest ART programs in the world, accounting for 20% of the global PLWHA. South Africa is among the first countries to adopt the new evidence-based policy of providing HIV treatment to all people who test HIV-positive as soon as possible without having to undergo additional tests such as CD4 cell count, to test the immune system [5,6]. Having started its nationwide roll-out of ART in 2004 and making ART freely available from all public health facilities, South Africa has about 60% of the estimated 5.4 million people that are registered on the ART program on first-line dolutegravir-based (DTG) regimen [7,8]. However, the country achieved only 61% viral suppression among people who were on ART in 2020, a figure that was less than the global 66%, in the same year [9].

Whilst several studies have cited non-adherence to treatment, acquired or transmitted resistance to ART, and challenges relating to pharmacokinetics such as poor absorption, drug interaction, or underdosing as the major hurdle in achieving viral suppression; effects of age of participants including gender, advanced HIV disease, and duration on ART among others, have also been cited [10-13]. The latter could be due to the length of time required for the treatment, which is life-long and daily in nature [14-17]. This study aimed to measure the proportion of patients who failed to achieve viral suppression following ART initiation, and to determine the factors associated with virological non-suppression.

## Methods

**Study design:** the study used a descriptive cross-sectional study and collected secondary data from the health facility records and conducted face-to-face interviews among a sample of HIV-positive patients who were accessing ART services at the selected primary healthcare facilities (PHCs) of Vulindlela sub-district of Pietermaritzburg in KwaZulu Natal (KZN) province.

**Study setting:** the setting for this study was the PHC facilities located in Vulindlela which is one of eleven sub-districts of uMgungundlovu district located in the province of KZN of South Africa. Vulindlela sub-district is situated in uMsunduzi local municipality of uMgungundlovu district in KZN, with an estimated 618,536 inhabitants, mostly isiZulu speaking. The Unemployment rate is 33%, characterized by high levels of poverty [18]. The sub-district is peri-urban, with tracts interspersed with settlements with limited services [19]. It is characterized by high HIV rates, 36.4%, among men and women aged 15-49 years old in 2018, a figure which was twice the average national prevalence of 18.8% [20]. The data collection process occurred between October 2022 and December 2022. Participants were recruited during their health facility visits, during which the purpose of the study was explained to potential participants. Those who agreed to participate were requested to sign the IC forms and were interviewed on the day after they had received the health service. Others opted to have the interviews on the next appointment date. During the interviews, the purpose of the study was re-explained and issues of anonymity and confidentiality, including risks and benefits, were discussed before the interviews could begin. The interviews took between 20 and 30 minutes to complete. The data were captured onto an Excel spreadsheet where these were cleaned and coded before importation for the statistical analysis.

**Data sources and target population:** we collected clinical data from the Tier net and used patient records to collect the socio-demographic and ART information. We also conducted face-to-face interviews to collect additional socio-demographic data, information on participant treatment taking as well as social and sexual behaviors. The self-administered and researcher-developed questionnaire was written in English and translated into isiZulu, which is the most spoken language in the study setting. The target population for this study was HIV-positive patients who had been initiated on ART between January 2017 and January 2019 at the selected PHCs in Vulindlela sub-district. Participants were included if they had been on ART for a least six (6) months, were 18 years old and above, and had given signed consent to participate. Patients who had been transferred out, including those who were lost to follow up were excluded from the study.

**Study size:** the sample size was determined using the Epicalculator (openepi.com). For the population size, we considered the 7,050 total number of HIV-positive patients who were initiated on ART between January 2017 and January 2019, and selected a 95% level of confidence, which corresponds to a 5% margin of error and chose a 50% response rate to obtain a minimum recommended sample size of 365. The final sample size was 401, after adding a 10% buffer. We then determined the percentage contribution of each of the four (4) selected health facilities to the total headcount and used these percentages to calculate the number of participants to be sampled from each health facility in proportion to the required sample size. The health facilities had been selected based on high headcount and ease of access.

**Participants:** study participants were selected using the systematic random sampling technique by selecting every nth patient name, after the health facility patient lists had been sorted alphabetically by name. The facility-specific nth value was determined by dividing facility-specific headcounts by the required facility-specific sample sizes [21].

**Variables:** the primary aim of this study was to measure the level of virological non-suppression and investigate associated factors. Virological non-suppression was dichotomized into yes and no categories and used as the outcome variable, and this was defined as having a viral load test result of at least 50 viral copies per milliliter of blood (>50 copies/ml blood), six (6) months following ART initiation. The sociodemographic and clinical variables were used as the explanatory variables, and these included age in years, sex, marital status, employment history, level of education, number of children, mode of transportation to a health facility, time taken to travel to a health facility, prior history of ART, type of ART regimen, and having comorbidity (ies).

Marital status was defined as whether the participant was married (cohabiting) or unmarried (single, divorced, separated, or widowed). Employment status was defined based on whether the participant was employed (full-time employment, part-time employment or self-employed) or unemployed (unemployed, pensioner, student). The level of education was defined based on the highest educational level attained by the participant. Prior history of ART was defined as whether the participant was ART naïve or ART experienced at the time of viral suppression assessment following the HIV diagnosis. Comorbidity was defined based on the recorded presence of any other disease or medical condition in addition to HIV infection. The study also collected treatment-taking and social and sexual behavior variables, and these included disclosure of a positive HIV status, missing treatment appointments, skipping treatment, knowing the partner's HIV status, and having multiple sexual partners, which was defined as having more than one (1) sexual partner at the time of HIV diagnosis and ART initiation.

**Data analysis:** for the analysis of data, the Excel data file was imported onto the Epiinfo 7 statistical software, where we conducted univariate analysis of the socio-demographic and clinical data. These were presented using descriptive statistics, with the numerical data presented as means and standard deviations (SD), and the categorical data presented as proportions and percentages. Frequency tables and figures were used to display these data. A chi-square test was used to determine the association between virological non-suppression and the study variables. A multivariate regression analysis was conducted to determine variables that were independently associated with virological non-suppression. The results were presented as adjusted odds ratios (aORs) with the corresponding 95% confidence intervals (95%CIs) and p values computed for statistical significance; p <0.05 and 95% CIs which exclude the null value of 1.

**Ethical approval and consent to participate:** the study obtained ethical clearance from the Research Ethics Committee of Sefako Makgatho Health Sciences University, reference number: SMUREC/H/219/2022: PG. Permission to conduct the study was granted by the KwaZulu-Natal Department of Health district office and the Provincial Health Research Committee of KwaZulu Natal, reference number: KZ-202208-011. Participants were requested to sign an informed consent form (ICF), if they were willing to participate after issues of voluntariness and withdrawal of participation at any stage of the study had been discussed.

## Results

**Socio-demographic characteristics of participants:** sixty percent of the study participants were female, (240/401). The mean age of the participants was 38 years (SD=11.1 years) with most aged between 29-39 years old accounting for 41.7% (167/401). The majority were single (346 (87.0%), unemployed, (216 (53.9%), and with a high school level of education (151 (41.1%)). More than half had ≤2 children, (241(60.1%)). Slightly more than half used a taxi when going to a health facility, (219 (54.4%), with 182 (45.4%) participants who took more than 1 hour to get to a health facility (Table 1).

**Table 1:** sociodemographic characteristics of participants (N=401)

Characteristic	Frequency	Percentage
**Sex**		
Female	240	60.0%
Male	161	40.0%
**Marital status**		
Single	346	87.0%
Married	50	12.5%
Divorced	3	1.0
Widowed	2	0.5
**Age (years)**		
Mean (SD)	38.1 (11.2)	
18-28	81	20.2%
29-39	167	41.7%
40-50	95	23.7%
51-61	39	9.7%
>61	19	4.7%
**Employment status**		
Employed	97	24.2%
Unemployed	216	53.9%
Pensioner	14	3.5%
Self employed	60	14.9%
Student	14	3.5%
**Education**		
Primary/ no schooling	76	20.7%
High school	151	41.1%
Tertiary	140	38.2%
**Number of children**		
<2	241	60.1%
≥2	160	39.9%
**Mode of transport**		
Taxi	218	54.4%
Walking	183	45.6%
**Time taken to travel to health facility**		
1 hour	219	54.6%
2 hours	167	41.7%
3 hours	15	3.7%
Total	401	100%

**Clinical details of the study participants:** the majority of the 401 participants were on regimen 1 (TLD (TDF/3TC/DTG), (383/95.5%). Most had never been on ART before (387(96.5%), with only 51 (12.7%) who had their medication collected by a treatment supporter. Comorbid conditions were observed among 24 (5.9%) of the 401 participants, most of whom had diabetes mellitus, 11/24 (45.8%), followed by epilepsy and hypertension, both 4 (16.7%) and 5 (2.8%) who had TB. The average number of pills that were taken daily by the 401 participants was 2 (SD=1.9), with 275 (68.6%) who took between 1 and 2 pills per day. Out of the 401 participants, (40(10%)) were virologically unsuppressed six (6) months post ART initiation (Table 2).

**Table 2:** clinical characteristics of participants (N=401)

Characteristics	Frequency	Percentage
**ART regimen**		
TLD (TDF/3TC/DTG)	383	95.5%
2NRTI+LPV/r	18	4.5%
**Previous history of ART**		
Yes	14	3.5%
No	387	96.5%
**Medication collected by supporter**		
Yes	51	12.7%
No	350	87.3%
**Comorbidity**		
Yes	24	5.9%
No	377	94.1%
**Comorbidity Type, n=24**		
Diabetes mellitus	11	45.8%
Epilepsy	4	16.7%
Hypertension	4	16.7%
Tuberculosis	5	20.8%
**Number of pills per day**		
Mean (SD)	2.0 (1.9)	
≤2	275	68.6%
3-4	117	29.2%
5-6	8	2.0%
>6	1	0.2%
**Virological non-suppression**		
Yes	40	10%
No	361	90%

**Figure 1:**
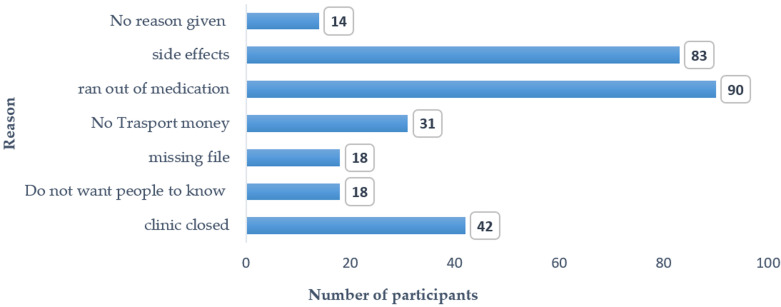
reason for stopping medication for more than 7 days (n=296)

**Treatment-taking and social behaviors of the study participants:** the treatment-taking behavior, and social and sexual behaviors of the study participants shows that only 51 (12.7%) of the 401 participants had their treatment collected by a treatment supporter. Most missed treatment appointments (335 (83.5%)), had ever skipped treatment (298 (74.3%)), had stopped taking treatment for >7 days (296 (74.2%)), and took prescription drugs (324 (80.8%)). Social and sexual behaviors revealed that the majority did not feel free to take their medication when there were people around (368 (91.8%)), missed treatment when there were people around (329 (97.3%)), and hid treatment (329 (97.8%)). Less than half (149 (37.2%)) had disclosed their positive HIV status, of which 60 (40.3%) reported having support after disclosure. Only 140 (34.9%) knew the HIV status of the partner, (35 (8.7%)) who reported having multiple sexual partners, and 68 (17%) that had drunk alcohol in the past three (3) months (Table 3). The reasons for stopping medication for >7 days as given by the 296 participants include, running out of medication was the most reported reason, (90/34%), followed by experiencing side effects (83 (28.0%)), clinic closed (42 (14.2%)), and no transport money (31 (10.5%)). Others stopped because the file was missing (18 (6.1%)), and others who did not want people to know that they were on medication (18 (6.1%)). Fourteen (14/296 (4.7%)) participants did not give any reason (Figure 1).

**Table 3:** treatment and social behaviors of participants (N=401)

	Frequency	Percentage
**Treatment taking behavior**		
**Collection of treatment by a treatment supporter (n=401)**		
Yes	51	12.7%
No	350	87.3%
**Missed appointment (n=401)**		
Yes	335	83.5%
No	66	16.5%
**Ever skipped treatment (n=401)**		
Yes	298	74.3%
No	103	25.7%
**Stopping treatment for >7 days (n=399)**		
Yes	296	74.2%
No	103	34.8%
**Taking non-prescription drugs (n=401)**		
Yes	324	80.8%
No	77	19.2%
**Feeling free taking medication when there are people around (n=401)**		
Yes	33	8.2%
No	368	91.8%
**Missing treatment when there are people around (n=338)**		
Yes	329	97.3%
No	72	21.3%
**Hiding treatment (n=401)**		
Yes	392	97.8%
No	9	2.2%
**Social and sexual behaviors**		
**Positive HIV status disclosure**		
Yes	149	37.2%
No	252	62.8%
**Support after disclosure (n=149)**		
Yes	60	40.3%
No	89	59.7%
**Knowing partners HIV status (n=401)**		
Yes	140	34.9%
No	261	65.1%
**Multiple sexual partners (n=401)**		
Yes	35	8.7%
No	366	91.3%
**Drinking alcohol in the past 3 months (n=401)**		
Yes	68	17%
No	333	83%

**Factors associated with virological non-suppression:** the univariate regression analysis revealed that the variables age of the participant, sex, employment status, number of children, time taken to travel to a health facility, prior history of ART, missing treatment appointment and skipping the treatment had statistically significant association with virological non-suppression. In multivariate analysis, the odds of viral non-suppression were higher among participants who were married, statistically significant, (aOR: 4.76, 95% CI 1.49-15.19; p = 0.008). The odds were lower among participants who: were male (aOR: 0.22, 95% CI 0.07-0.65; p=0.006), unemployed (aOR: 0.07, 95% CI 0.01-0.32; p=<0.001), had ≤2 children (aOR: 0.37, 95% CI 0.14-0.96; p=0.04), travelled for ≤1 hour to a health facility (aOR: 0.22, 95% CI 0.06-0.77; p = 0.02), had been on ART before (aOR: 0.01, 95% CI 0.003-0.08; p = <0.001), and those who were on ART regimen 2 (aOR 0.16, 95% CI 0.03-0.96; p=0.04), (Table 4).

**Table 4:** odds of virological non-suppression among participants initiated on ART between January 2017 and January 2019 at the selected PHCs in Vulindlela sub-district (n=401)

	Virological non-suppression			
	Unadjusted ORs (95% CI)	P value	Adjusted ORs (95% CI)	P value
**Age (years)**				
18-28	6.92 (1.40-34.14)	0.01	2.78 (0.36-21.4)	0.33
29-39	2.35 (0.70-7.90)	0.17	1.10 (0.23-5.42)	0.90
40-50	2.04 (0.57-7.25)	0.27	1.00 (0.19-5.38)	0.99
51-61	1.81 (0.43-7.72)	0.42	2.26 (0.32-16.15)	0.42
**Sex**				
Male	0.64 (0.33-1.23)	0.18	0.22 (0.07-0.65)	0.006
**Marital status**				
Married	2.67 (1.22-5.88)	0.01	4.75 (1.49-15.19)	0.008
**Employment history**				
Unemployed	0.19 (0.07-0.51)	<0.001	0.07 (0.02-0.31)	<0.001
**Level of education**				
Primary/no schooling	0.59 (0.30-1.17)	0.13	0.94 (0.31-2.80)	0.91
**Number of children**				
≤2	0.47 (0.22-0.99)	0.04	0.37(0.14-0.96)	0.04
**Travel time to a health facility**				
≤ 1 hour	0.18 (0.08-0.45)	<0.001	0.22 (0.06-0.77)	0.01
**Prior history of ART**				
Yes	0.01 (0.006-0.08)	<0.001	0.01 (0.003-0.08)	<0.001
**Type of ART regimen**				
Regimen 2 (2NRTI+LPV/r)	0.36 (0.11-1.16)	0.08	0.16 (0.03-0.96)	0.04
**Comorbidity**				
Yes	0.39 (0.14-1.11)	0.08	0.52(0.09-2.76)	0.45
**Disclosure of positive HIV status**				
Yes	1.63 (0.79-3.37)	0.19	1.06(0.41-2.71)	0.90
**Missed treatment appointments**				
Yes	0.12 (0.01-0.86)	0.03	0.38 (0.08-1.75)	0.21
**Skipped treatment**				
Yes	0.13 (0.03-0.57)	0.006	0.29 (0.06-1.42)	0.13
**Mode of transportation**				
Walking	1.15 (0.59-2.23)	0.67	-	-
**Number of pills/daily**				
≤2	0.70 (0.33-1.49)	0.36	-	-
**Knowing partner′s HIV status**				
Yes	0.69 (0.36-1.36)	0.29	-	-
**Having multiple sexual partners**				
Yes	1.91 (0.44-8.28)	0.39	-	-

## Discussion

This study determined the level of virological non-suppression among ART participants and found 10% of participants that were virologically unsuppressed, post-ART initiation. This finding is consistent with reported levels of virological non-suppression from other studies, ranging between 6.1% and 8.7% [22,23]. However, others have reported much higher levels of virological non-suppression ranging between 20.3% and 97.2% of ART participants on DTG-based regimens [24-27]. The study found participants who had never been on ART before to constitute 72% of those who were virologically unsuppressed. Similarly, others have reported higher virological non-suppression among participants who had been on ART for shorter periods on ART [23]. However, reports of virological non-suppression have been cited among participants who have been on ART for longer periods [26,28]. The observed self-reported non-disclosure of positive HIV status, including behaviors of missing treatment appointments, and skipping and stopping taking treatment for more than 7 days observed among participants in this study have been reported in other studies [29-31]. This study further investigated the factors associated with virological non-suppression and found higher odds among participants who were married compared to those who were unmarried. Similarly, a study conducted in Borno State of Nigeria also reported low viral suppression among married participants [32].

The finding of 10% virological non-suppression is an equivalence of 90% viral suppression, a figure which falls short of the 95% target of the UNAIDS strategy of 95-95-95. The finding of 72% ART inexperienced participants among the 10% that were virologically unsuppressed is worrisome because virological non-suppression has been linked with long lengths of time on ART [23]. The observed non-disclosure of positive HIV status could be a result of fear as disclosure has been associated with such negative consequences as partnership dissolution, abandonment, and partner violence, especially among the female participants [33-35], however, disclosure should be encouraged. The finding of an association between marital status and virological non-suppression could be an indication of a lack of spousal support or non-disclosure of positive HIV status [36,37].

**Limitations:** the present study had limitations, and these include the small sample size, which led to the observed wider 95% confidence intervals. In addition, the medical records did not include measures of adherence such as pill counts or on-time drug pickups. The study also did not include all ART patients who were on the Central Chronic Medicines Dispensing and Distributing (CCMDD) programme, which could lead to an underestimated level of viral non-suppression.

## Conclusion

The observed virological non-suppression of 10%, and which translates to 90% viral suppression, indicates the amount of effort that is needed to help achieve or sustain viral suppression. This needs to be addressed especially if the WHO target of ending HIV/AIDS by 2030 is to be realized. Of particular concern is the finding of the 72% ART naive participants among the 10% participants that were virologically unsuppressed; virological non-suppression has been associated with lengthy periods of time on ART. Pre-ART counselling is meant to help HIV-positive patients make well informed decision to test for HIV and commit to ART medication which is life-long in nature. Non-disclosure of positive HIV status and the associated social behaviors highlight the role of stigma and discrimination which PLWHA continue to experience in the communities.

### What is known about this topic

- *Virological non-suppression is common among patients who are ART experienced, this is presumably a result of pill fatigue due to the life-long nature of ART*;
- *Disclosure of positive HIV status improves adherence to ART, which is a well-established predictor of viral suppression*.

### What this study adds

- *Virological non-suppression was higher among patients who were ART naïve*;
- *Non-disclosure of positive HIV status continues to hinder successful treatment outcomes such as viral suppression, and therefore efforts should be made to encourage disclosure*.
